# Effector Avr4 in *Phytophthora infestans* Escapes Host Immunity Mainly Through Early Termination

**DOI:** 10.3389/fmicb.2021.646062

**Published:** 2021-05-28

**Authors:** Abdul Waheed, Yan-Ping Wang, Oswald Nkurikiyimfura, Wen-Yang Li, Shi-Ting Liu, Yahuza Lurwanu, Guo-Dong Lu, Zong-Hua Wang, Li-Na Yang, Jiasui Zhan

**Affiliations:** ^1^Key Lab for Bio Pesticide and Chemical Biology, Ministry of Education, Fujian Agriculture and Forestry University, Fuzhou, China; ^2^Department of Crop Protection, Bayero University Kano, Kano, Nigeria; ^3^Key Laboratory of Ecological Pest Control for Fujian and Taiwan Crops, Fujian Agriculture and Forestry University, Fuzhou, China; ^4^Institute of Oceanography, Minjiang University, Fuzhou, China; ^5^Department of Forest Mycology and Plant Pathology, Swedish University of Agricultural Sciences, Uppsala, Sweden

**Keywords:** *Phytophthora infestans*, effector gene, mutation mechanism, population genomics, protein truncation, thermal adaptation

## Abstract

Effector genes play critical roles in the antagonistic interactions between plants and pathogens. However, knowledge of mutation mechanisms and evolutionary processes in effector genes and the contribution of climatic factors to the evolution of effector genes are fragmented but important in sustainable management of plant diseases and securing food supply under changing climates. Here, we used a population genetic approach to explore the evolution of the Avr4 gene in *Phytophthora infestans*, the causal agent of potato blight. We found that the Avr4 gene exhibited a high genetic diversity generated by point mutation and sequence deletion. Frameshifts caused by a single base-pair deletion at the 194th nucleotide position generate two stop codons, truncating almost the entire C-terminal, which is important for effector function and R4 recognition in all sequences. The effector is under natural selection for adaptation supported by comparative analyses of population differentiation (*F_ST_*) and isolation-by-distance between Avr4 sequences and simple sequence repeat marker loci. Furthermore, we found that local air temperature was positively associated with pairwise *F_ST_* in the Avr4 sequences. These results suggest that the evolution of the effector gene is influenced by local air temperature, and the C-terminal truncation is one of the main mutation mechanisms in the *P. infestans* effector gene to circumvent the immune response of potato plants. The implication of these results to agricultural and natural sustainability in future climate conditions is discussed.

## Introduction

Plants, with an array of defense machinery (immunity), are overwhelmed by a diverse group of pathogens, each deploying with a series of invasive mechanisms (Lamour and Kamoun, 2009; Zhan et al., 2015). The dynamics of these defense and invasive processes takes place in a co-evolutionary arms race (McDonald and Linde, 2002; Zhan et al., 2014) in which current defense mechanisms in plants select for novel pathogen’s invasive systems that reduce the efficacy of existing defenses and trigger the emergence of new defense (Wu et al., 2016). This model of host-pathogen interaction was elucidated more than 70 years ago (Flor, 1942), but its molecular basis has been poorly understood until the 2000s (Jones and Dangl, 2006; Thrall et al., 2016; Yin et al., 2017). It involves a group of proteins called effectors that trigger the plant defense system (hypersensitive response) when they are detected by receptor proteins produced by host resistant (R) genes (Hein et al., 2009). In addition to being the binding targets of resistant receptors, effector proteins also play crucial roles for the survival, infection, and proliferation of many pathogens (Van Der Biezen and Jones, 1998; Wang et al., 2015; Tanaka et al., 2019), such as manipulating cellular procedures of host plants toward higher susceptibility and constraining the production of compounds involved in plant immune systems. For example, the Avr4 protein secreted by *Cladosporium fulvum* binds with chitins to protect the pathogen against plant chitinases (Van Der Biezen and Jones, 1998).

Genomic analysis reveals many effectors exist in plant pathogens. According to their cellular localizations and pathways of attacking host plants, these effectors can be divided into two categories: apoplastic effectors and cytoplasmic effectors. Apoplastic effectors suppress host immunity by interfering with plant extracellular compounds such as proteases and glucanases (Kamoun, 2006; Tian et al., 2007; Schornack et al., 2009). Cytoplasmic effectors are recognized by plant receptors such as nucleotide-binding leucine-rich repeat, resulting in programming host cell death (hypersensitive reaction; Birch et al., 2009). Some cytoplasmic effectors also play roles in promoting pathogen colonization, suppressing host basal resistance, or inducing host plant susceptibility (Kamoun, 2006; Asai and Shirasu, 2015). Due to their importance in pathogen adaptation to the rapid and constant change of defense in plant hosts, effector genes are expected to evolve more rapidly compared with other parts of pathogen genomes. Many effector genes occupy a long intergenic region surrounded by gene-sparse and transposon-rich genome (Tyler et al., 2006; Harry et al., 2009). This physical location provides effector genes a better opportunity to generate mutations. Indeed, genomic and functional studies have discovered multiple genetic mechanisms including base substitution, insertion, deletion, pseudogenization, and transcriptional silencing are involved in the evolution of effector genes (Shan et al., 2004; Win et al., 2007; Wang et al., 2019a).

Many factors can affect the population genetic structure and evolution of pathogen effectors. In addition to genetic characteristics of associated pathogens, climatic factors such as temperature may also manipulate the population genetic dynamics and evolutionary pathway of effector genes. As an omnipotent force, temperature regulates the population genetic structure and evolution of effector genes through its impacts exerting on all aspects of biotic and abiotic activities in nature such as thermodynamics of nucleotide, genetics, physiology, survival and reproduction of plants and pathogens, and interaction of pathogens with their hosts and community. In the context of global warming, information related to these temperature impacts is important not only to understand the evolution of effector genes but also to address social concerns on future food security, human health, and ecological sustainability (Velásquez et al., 2018). Indeed, it has been documented that temperature can affect genetics and quasi-genetics of effector genes such as its genetic variation, gene expression, and competition through which influence efficacy and durability of plant resistance and food production (Banta et al., 1998; Martin, 2000; Mboup et al., 2012; Menna et al., 2015). In the *Arabidopsis thaliana-Puccinia striiformis* interaction, plants activate effector-triggered immunity systems at relatively low temperatures but adopt pattern-triggered immunity systems at higher temperatures (Cheng et al., 2013). Temperature also affects the pathogenicity, expression, and/or spatial distribution of effector genes in pathogens such as *Pseudomonas syringae*, *Pythium aphanidermatum*, *Pythium deliense*, and *Phytophthora infestans* (Martin, 2000; Mboup et al., 2012; Menna et al., 2015).

Oomycete *P. infestans* (Mont) de Bary causes severe disease in potatoes and tomatoes (Fry, 2008). It is the major constraining factor of potato production in many parts of the world, including China, the largest potato producer on the planet with an annual yield of 95.5 million tonnes (Faostat, 2017). The annual economic loss caused by this pathogen in potato alone is ~170 billion USD globally and with ~40 billion USD from China (Bos et al., 2003; Wu et al., 2012). *P. infestans* can reproduce asexually and sexually by outcross between two genotypes with comparable mating types (A1 and A2) or selfing of self-fertile genotypes (Zhu et al., 2016) and is featured by rapid evolution capable of defeating potato resistance quickly. Its genome consists of >550 effector genes and has rich repetitive sequences situating around the effector genes (Haas et al., 2009). A broad spectrum of effectors in the pathogen belongs to the RxLR class carrying an N-terminal signal peptide followed by a conserved Arginine-X-Leucine-Arginine (RxLR)-dEER domain and a C-terminal usually comprised W-Y-L motifs (Jiang et al., 2008).

In the past two decades, several *P. infestans* effector genes, including Avr4 (PITG_07387), have been functionally and structurally characterized. Avr4 is an RxLR gene consisting of virulent and avirulent alleles. Its avirulent alleles are translated into effector proteins recognized by the corresponding R4 protein in potato plants (Van Poppel et al., 2009). The total length of the wild-type avirulent Avr4 protein has 288 amino acids (aa) comprising an N-terminal signal peptide followed by an RxLR-dEER, three W motifs, and a Y-motif (Van Poppel et al., 2008, 2009). Frameshifts caused by two single-base deletions (ΔT^12^ and ΔT^196^) generate truncated proteins that transfer Avr4 from avirulent to virulent form. Functional analyses demonstrate that the deletion of the RxLR-dEER domain neither enhances nor suppresses the elicitor activity of the Avr4 proteins (Van Poppel et al., 2008). W2 motif is essential for the elicitor activity of the effector, but the full function of the effector requires the presence of Y-motif together either with the W1 or W3 domain (Van Poppel et al., 2009).

Effector research has historically focused on molecular and functional characterization, cellular localization, and signal transduction (Vleeshouwers et al., 2011; Goritschnig et al., 2012; Wang et al., 2019b). This research is important to understand the physical structure of effector proteins and the roles of individual domains in regulating pathogen pathogenicity, host plant immune response, biochemical processes, and cascades involved in the regulations. Fewer studies have been dedicated to exploring mutation mechanisms and evolutionary processes shaping effector genes and/or the contribution of climatic factors to the evolution by population genetic analysis of their sequence variation and spatial distribution in the pathogens. Hence, the specific objectives of the current study were to (i) analyze the population genetic structure of the Avr4 gene by sequence analysis of 114 *P. infestans* isolates originating from several spatial regions of China varying in thermal condition, (ii) determine mutation mechanisms creating the genetic variation of the effector, (iii) determine the relative contribution of natural selection and genetic drift on the population genetic structure and evolution of the Avr4 gene, and (iv) infer the role of air temperature in the local adaptation of effector genes.

## Materials and Methods

### *Phytophthora infestans* Collection and Isolation

**Figure 1:**
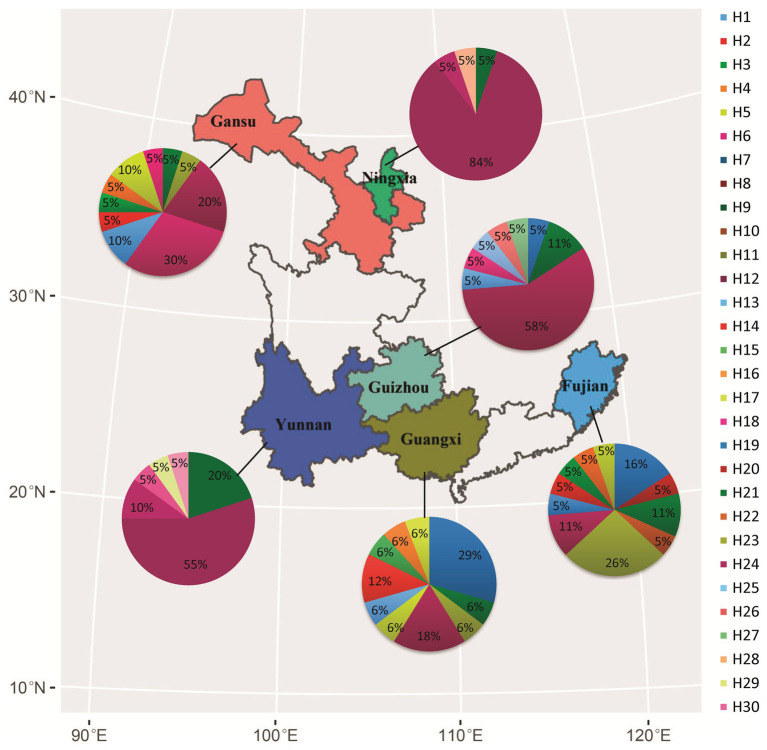
Geographical locations of collection sites, frequency, and spatial distribution of nucleotide haplotypes in the Avr4 gene of *P. infestans* sampled from six fields in China.

A total of 114 *P. infestans* isolates selected from Fujian, Gansu, Guizhou, Guangxi, Ningxia, and Yunnan collections were included in the current study (Table 1; Figure 1). The isolates were derived from potato leaves infected with late blight pathogen in the six locations during cropping seasons between 2010 and 2012. The leaves in the same location were sampled on the same day from different plants in the same field. Each leaf sample was packaged separately in plastic bags and transferred in an icebox to the laboratory within 24 h for pathogen isolation. After cleaned by running tap water and sterilized distilled water, the infected leaves were placed abaxial side up in Petri dishes filled with 2% water agar and then incubated at 18°C under dark conditions for 24–48 h to stimulate sporulating. The pathogen was isolated by peeling off a piece of mycelium from a sporulating lesion and transferred to a rye B plate amended with 100 μg/ml ampicillin using an inoculating needle. The isolates were purified by two to three consecutive transfers of single mycelium to new plates and stored in a cold room until use. Genotypes of the isolates were determined by molecular amplification of simple sequence repeat (SSR) markers (Knapova and Gisi, 2002; Lees et al., 2006), restriction enzyme-PCR amplification of mitochondrial haplotypes (Flier et al., 2003), mating type (Zhu et al., 2015), and partial sequence analysis of b-tubulin, Cox1, and Avr3a (Cárdenas et al., 2011). Detailed information on pathogen collection, isolation, and genotyping can be found in our previous publications (Qin et al., 2016).

**Table 1:** Sample size and sequence variation of Avr4 gene in the *P. infestans* populations collected from six locations in China and their correlation coefficients with annual mean temperature (AMT) at the collection sites.

Collection site	AMT (°C)	Number of sequences	Polymorphic sites	Number of haplotypes	Haplotype diversity	Nucleotide diversity
Fujian	20.5	19	11	11	0.912	0.003
Gansu	11.7	20	11	10	0.879	0.002
Guizhou	14.7	19	7	8	0.673	0.002
Guangxi	22.6	17	7	10	0.897	0.003
Ningxia	7.0	19	3	4	0.298	0.001
Yunnan	15.6	20	4	6	0.674	0.001
Pooled		114	19	30	0.808	0.002
Correlation			0.402 (0.43)^a^	0.718 (0.11)	0.782 (0.07)	0.804 (0.05)

a Correlation coefficient and its corresponding *p*-value (in parenthesis).

### Avr4 Sequencing

*Phytophthora infestans* isolates retrieved from long-term storage were cultured on rye B agar at 18°C under dark conditions for 2 weeks. Only isolates with distinct genotypes were selected for sequence analysis of the Avr4 gene. As a result, 114 *P. infestans* isolates with 17–19 isolates from each of the six populations were included in the sequence analysis of the Avr4 gene (Table 1). Genomic DNAs of the isolates were extracted from the lyophilized mycelia (~100 mg/isolate) with gDNA Kit (Pomega Biotch. Co. Ltd., Beijing) following the standard protocol provided by the manufacturers with some minor modifications and were amplified by a pair of Avr4 specific primers (PiAvr4 For: ATGCGTTCGCTTCATTTTGCTGG, and PiAvr4 Rev.: CTAAGATATGGGCCGTCTAGCTTGGAG) as reported previously (Van Poppel et al., 2008). PCR amplifications of the Avr4 gene were performed in a 25-μl reaction buffer composed of a 2.5-μl 10× PCR buffer (MG^2+^ free), 2-μl deoxynucleoside triphosphate (2.5 mmol/L), 1-μl PiAVR4F (10 μmol/L), 1-μl PiAVR4R (10 μmol/L), 1-μl template DNA, 17.3-μl double-distilled water, and 0.2-μl (5 U/μl) Taq DNA polymerase (Trans Gene Biotech Co., Ltd., Beijing, China) using Gene Cycler TM (Bio-Rad). The PCR amplifications were started by 94°C DNA denaturation for 2 min, followed by 35 cycles of 1 min (amplification) at 95°C, 1-min annealing at 56°C, and 1.5-min extension at 72°C and ended with a further 5-min extension at 72°C (Van Poppel et al., 2008). PCR samples were loaded on gel electrophoresis (1%) and purified for single direction sequencing as suggested by the manufacturer (QIA quick® Gel Extraction Kit). The products were ligated to T5 zero cloning vector and transformed with *Trans*1-T1 into Competent cells by the heat-shock process at 42°C for 30 s (pEASY®-T5 Zero Cloning Kit). Three colonies were randomly picked from each transformation and incubated in Luria-Bertani liquid media at 37°C overnight under continuous shaking. One colony was selected and sequenced by Gene Script Biological Technology Co., Ltd. (Gene Script, Nanjing, China) using an ABI3730 automated DNA sequencer (Applied Bio-systems, United States).

### Data Analysis

All nucleotide sequences of the Avr4 gene were visually assessed to remove possible fake “mutations” generated by PCR artifacts (Zhou et al., 2019). A reference Avr4 sequence (ID: KF188223) was downloaded from National Center for Biotechnology Information (NCBI). MUSCLE embedded in the software MEGA5 was used to perform the codon-based algorithm alignment according to the reference sequence, and population diversity parameters, including variation sites, the number of haplotypes (h), haplotype diversity (HD), and nucleotide diversity (π), were estimated (Tamura et al., 2011). Genetic differentiation (*F_ST_*) in Avr4 between and among different *P. infestans* populations was analyzed using Arlequin V3.5 (Excoffier and Lischer, 2010) and was tested by Hudson’s permutation with 1,000 replicates (Librado and Rozas, 2009).

SSR data of the 114 isolates were taken from a previous publication (Wu et al., 2016). POPGENE version 1.32^1^ was used to calculate SSR differentiation between pairs of the populations and among the populations according to fixation index (*F_ST_*). The percentile of the SSR *F_ST_* was generated by bootstrapping method with 100 replications of the original data with Resampling 6.20 as reported (Zhan et al., 2003). Evolutionary history in the Avr4 gene was evaluated by a two-tailed t-test by comparing the population differentiations between the Avr4 gene and SSR marker loci (Gao et al., 2017). It is expected that selection for local adaptation will lead to a significantly higher Avr4 *F_ST_* than SSR *F_ST_*. Lower *F_ST_* in the Avr4 gene than SSR marker loci will be generated by a global selection for the same Avr4 mutations over all spatial populations, whereas a similar level of the two measurements indicates the neutral evolution of the Avr4 gene (Wu et al., 2016). A median-joining network was created and displayed to demonstrate the genetic relationship among the nucleotide haplotypes using PopArt software.^2^ Isolation by distance was evaluated by conducting a Pearson correlation analysis (Lawrence and Lin, 1989) between the pairwise *F_ST_* in the Avr4 gene or SSR marker loci and the geographical distance in kilometers. Similarly, the impact of air temperature in the collection sites on the nucleotide variation and its spatial distribution of the Avr4 gene was evaluated by the Pearson correlation analysis described previously (Yang et al., 2016). The geographic distance between the collection sites was calculated using the geographic coordinates of sites from which the *P. infestans* populations were sampled. Annual mean temperature in the collection sites was downloaded from World Climate^3^ and was presented as an average over 15–30 years.

## Results

### Sequence Variation in the Avr4 Gene

**Figure 2:**
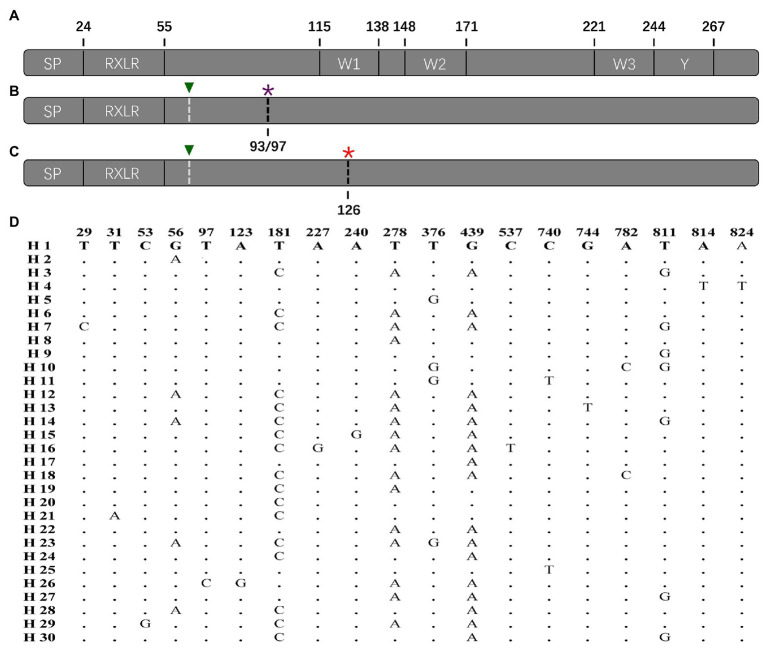
Protein structure of *P. infestans* Avr4 effector deduced from the reference sequence and nucleotide structure of the Avr4 gene generated from the current study: **(A)** primary protein structure of reference isolate (KF188223) containing a signal peptide (SP), a conserved RxLR-dEER domain, three W (W1, W2, and W3) motif, and a conserved Y motif; **(B)** the structure of Avr4 gene with early terminations caused by the 194th nucleotide deletion. The 194th nucleotide deletion showed by white dashed line with green triangle and the stop codon caused by the 194th nucleotide deletion showed by the black dashed line with a purple asterisk; **(C)** structure of Avr4 gene with a potential early termination caused by the 376th point mutation after the 194th nucleotide deletion. The 194th nucleotide deletion showed by white dashed line with green triangle and the stop codon caused by the 376th point mutation showed by a black dashed line with a red asterisk; **(D)** polymorphic sites in the 30 nucleotide haplotypes of the Avr4 gene.

The complete Avr4 in the reference sequence (KF188223) downloaded from NCBI contains 867 nucleotides, which are translated to a protein with 287 amino acids [excluding start and stop codons (Figure 2A)]. The Avr4 protein consists of a signal peptide (SP), a conserved RxLR-dEER domain, three conserved W domains (W1, W2, and W3), and the Y motif (Figure 2A). None of the current 114 sequences matches the reference sequence (KF188223). Nineteen variable sites were identified in the 114 sequences, generating a total of 30 nucleotide haplotypes (Figure 2D). A base pair deletion (ΔT^194^) was detected in all of the 114 sequences, generating two stop codons to truncate the entire C-terminal of the effector protein starting from the 93rd amino acid (33 sequences) or 97th amino acid (81 sequences; Figure 2B). Alignment also showed another potential stop codon generated by a point mutation in 376th nucleotide (G376T) that was found in 104 (91%) of the untranslated parts of the nucleotide sequences (Figure 2C). The 10 sequences without the point mutation were genetically closer to the reference downloaded from NCBI (Figure 2B).

Nucleotide diversity in the six populations ranged from 0.001 to 0.003 with a grand mean of 0.002 when sequences from different populations were pooled together. The highest nucleotide diversity was found in Fujian and Guangxi populations, whereas the lowest nucleotide diversity was detected in Ningxia and Yunnan populations (Table 1). Among the 30 haplotypes, H6 was detected 47 times and was the most dominant in the pooled population, followed by H3, which was detected 11 times. These two haplotypes were found in all six populations (Figure 1). H6 was also the dominant haplotype in populations from Ningxia, Guizhou, and Yunnan with a frequency of 84, 58, and 55%, respectively.

H1, H5, and H12 were detected in a total of nine, seven, and nine times from four (Fujian, Gansu, Guangxi, and Guizhou), three (Fujian, Gansu, and Guangxi), and three (Gansu, Ningxia, and Yunnan) populations and were the dominant haplotypes in populations from Guangxi, Fujian, and Gansu with a frequency of 29, 26, and 30%, respectively. Although H17 was detected three times and H13, H19, H20, and H24 were detected two times, all of them were private to one of the populations. The remaining 20 haplotypes were only detected once (Figure 1). Haplotype diversity in the six populations ranged from 0.298 to 0.912 with a grand mean of 0.808 when isolates from individual populations were pooled (Table 1). The highest haplotype diversity and richness were found in the Fujian population, and the lowest haplotype diversity and richness were found in the Ningxia population. The annual mean temperature in the sample collection sites was positively associated with sequence variation in the Avr4 gene but only significant with nucleotide diversity (Table 1, *p* = 0.05).

### Population Genetic Differentiation in the Avr4 Gene and Neutral Simple Sequence Repeat Loci

**Figure 3:**
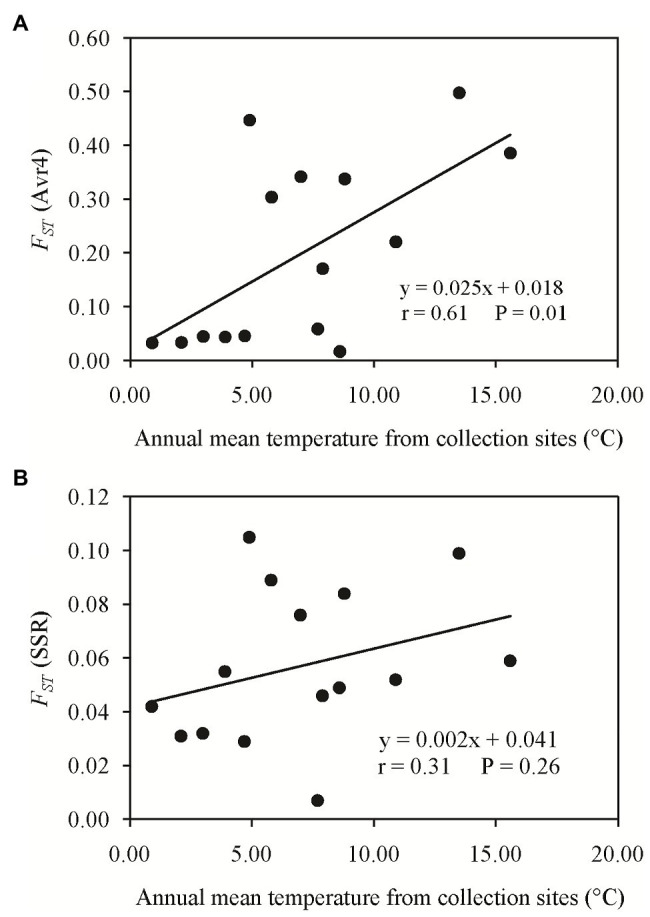
Association of the absolute difference in air temperature between collection sites and the pairwise *F_ST_*: **(A)** Avr4 gene and **(B)** SSR marker loci.

Pairwise *F_ST_* in the Avr4 gene estimated from nucleotide frequencies ranged from 0.017 to 0.498 with an average of 0.199, and pairwise *F_ST_* in neutral SSR loci ranged from 0.007 to 0.105 with an average of 0.057 (Table 2). Nine pairs of *F_ST_* in the Avr4 gene were significantly higher than the corresponding pairwise *F_ST_* in SSR loci. The overall *F_ST_* in the Avr4 gene was 0.252, which was significantly higher than the overall *F_ST_* in SSR marker loci (*p* = 0.001). The difference in air temperature between collection sites was positively associated with pairwise *F_ST_* in the Avr4 gene and SSR marker loci, but only the association with the *F_ST_* in the Avr4 gene was significant (Figure 3).

**Table 2:** Pairwise population differentiation (*F_ST_*) in SSR marker loci (above diagonal) and Avr4 gene (below diagonal).

	Fujian	Gansu	Guangxi	Guizhou	Ningxia	Yunnan
Fujian	–	0.084	0.031	0.089	0.099	0.105
Gansu	**0.338**	–	0.052	0.032	0.029	0.055
Guangxi	0.034	**0.221**	–	0.046	0.059	0.076
Guizhou	**0.304**	0.045	**0.171**	–	0.007	0.042
Ningxia	**0.498**	0.046	**0.386**	**0.059**	–	0.049
Yunnan	**0.447**	0.044	**0.342**	0.033	0.017	–

Bold font indicates that AVR4 F_ST_ is greater than SSR F_ST_ in the pair of *P. infestans* populations.

### Haplotype Network and Isolation by Distance

**Figure 4:**
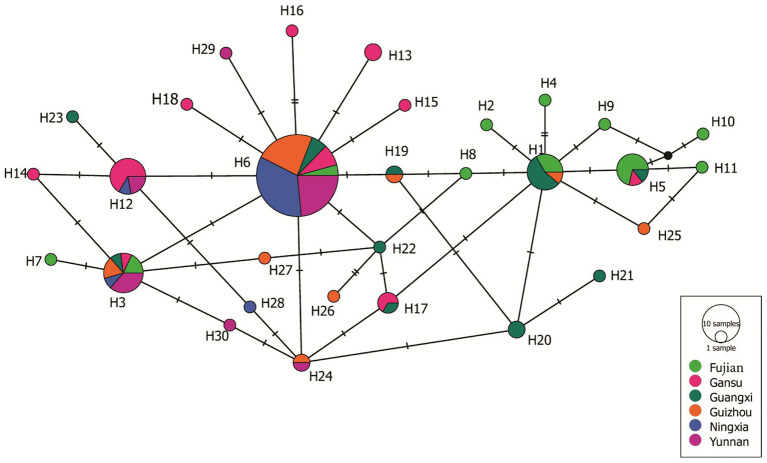
Haplotypes network of the 30 Avr4 nucleotide haplotypes generated from the six *P. infestans* populations in China. Colors represent geographic origins (populations) of the haplotypes, and circle sizes represent haplotype frequency in the populations.

**Figure 5:**
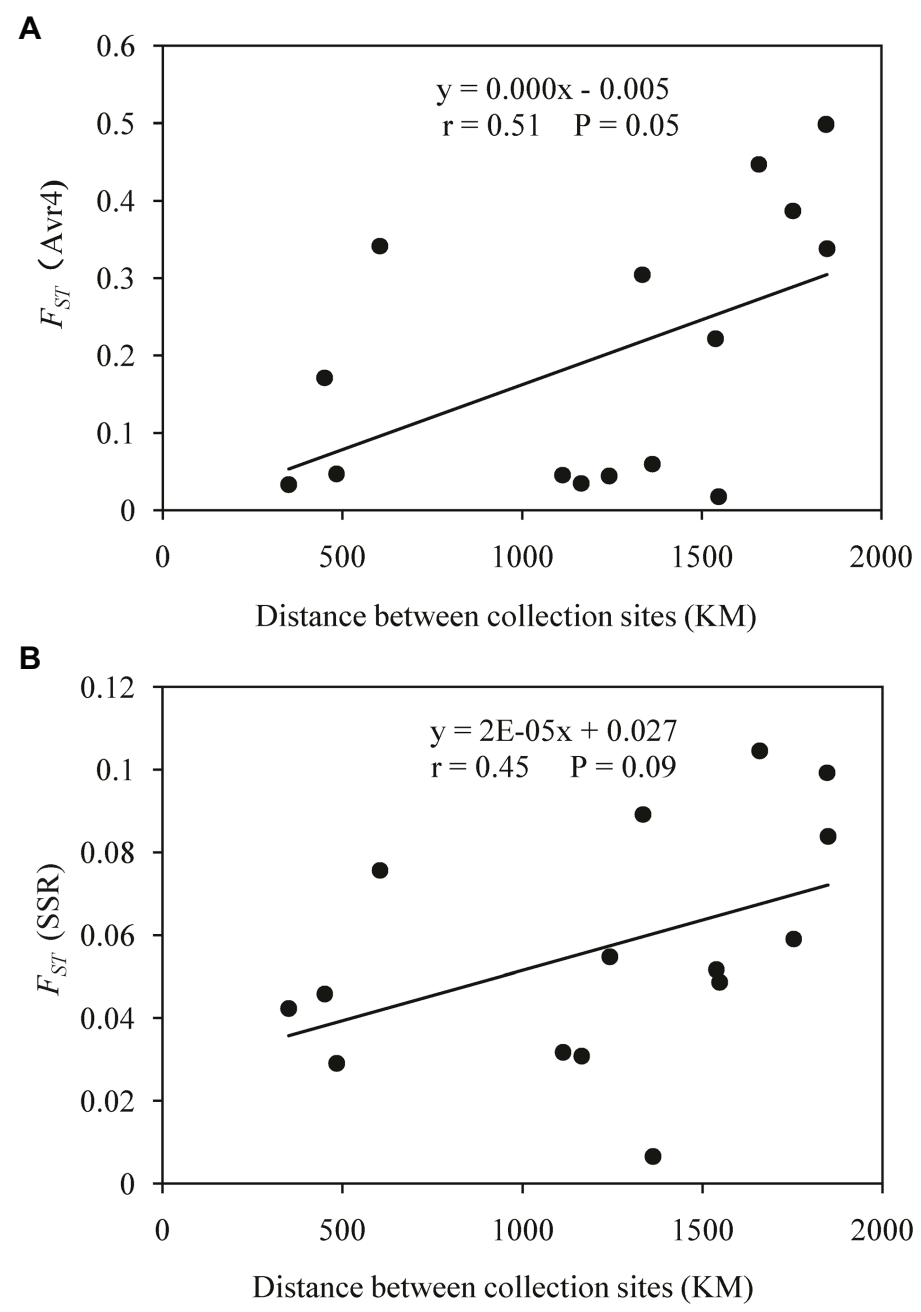
Isolation by distance analysis (IBD) of genetic differentiation in the Avr4 and SSR marker loci of *P. infestans* populations sampled from China: **(A)** *F_ST_* of the Avr4 gene and **(B)** *F_ST_* of the SSR marker loci.

A haplotype network was generated from 19 single nucleotide polymorphic sites of the 30 haplotypes. This analysis revealed that most of the haplotypes were two to three mutation steps away from each other. With 10 mutation steps, H10 was genetically farthest from the dominant H6 (Figure 4). Most haplotypes within the same population were clustered together. For example, in the 11 haplotypes detected in Fujian (light green), H1, H2, H4, H5, and H8–H11 were clustered together except H3, H6, and H7, and the nine haplotypes in the Guangxi population (dark green) were clustered together except H23, which was clustered together with the haplotypes from other populations. Fujian was the most diverse population in the samples. It consisted of haplotypes genetically most distant from each other and from the dominant haplotype H6. For example, there were 16 mutation steps (farthest) between H7 and H10 and 14 mutation steps between H7 and H11. The genetic distance between H6 and H11 was 10 mutation steps. On the other hand, Ningxia was the least diverse population. Only four haplotypes were detected in the population with six mutation steps between the two genetically most distant haplotypes (H3 and H28). The 19 haplotypes were linked by many reticulation structures (Figure 4). Isolation by distance analysis showed that geographic distance was positively and significantly associated with the *F_ST_* in the Avr4 gene (Figure 5A) but not with the *F_ST_* in the SSR marker loci (Figure 5B).

## Discussion

According to our best knowledge, this is the first attempt to understand the population genetic structure, mutation mechanisms, and evolutionary processes of *P. infestans* Avr4 gene and its interaction with air temperature using a large collection of isolates sampled from various ecological niches. We found a high genetic diversity in the Avr4 gene. A total of 30 nucleotide haplotypes were observed from the 114 sequences. This level of genetic diversity is comparable with other effector genes, including Avr3a, Avr2, and AvrSmira1 but higher than other functional genes such as eEF-1α, cox1, and β-tubulin genes of *P. infestans* (Cárdenas et al., 2011; Stefańczyk et al., 2018; Yang et al., 2018; Wang et al., 2020). This result is consistent with evolutionary theory postulating that genomes involved in antagonistic interactions such as effector genes tend to evolve at an increased speed (Stukenbrock and McDonald, 2009; Karasov et al., 2014; Möller and Stukenbrock, 2017).

Sequence alignment indicates that base substitution is the main mechanism creating the sequence polymorphism in the Avr4 gene (Figure 2). Although 19 SNP sites formed only 30 haplotypes, many of them are separated by more than 10 mutation steps (Figure 4). Although many reticulation structures were found from the haplotype network analysis, no recombination event was detected in the gene by any of the seven algorithms (data not shown) integrated into the RDP4 programs (Martin et al., 2015), suggesting that the reticulation structures in the Avr4 gene were likely generated by convergent mutation rather than nucleotide reshuffling among the existing sequences. In addition to base substitution, the nucleotide deletion (ΔT^194^) was also detected in the gene. However, two single-base deletions (ΔT^12^ and ΔT^196^) reported previously (Van Poppel et al., 2008) were not found in the current study, suggesting spatial heterogeneity in the gene.

The highest sequence variation was found in the *P. infestans* population from Fujian but lowest from Ningxia, Guizhou, and Yunnan. Ningxia, Guizhou, and Yunnan are among the top potato production regions in China, with >0.6 million annual hectares in each of the regions, whereas Fujian only grows ~1/10 acreage of each of the regions. The finding of the highest genetic variation in the *P. infestans* Avr4 gene from Fujian is unexpected but consistent with previous results derived from other effector genes (e.g., Avr2 and AVR3a), phenotypic traits (fungicide resistance and aggressiveness), and ecological data (e.g., growth rate and ultraviolet tolerance) of the pathogen (Qin et al., 2016; Yang et al., 2018, 2020; Wu et al., 2019, 2020; Lurwanu et al., 2021). Unlike Guizhou, Ningxia, Yunnan, and many other regions where well-established seed production systems can ensure the adequate supply for own use, potato in Fujian relies on imported seeds from other parts of China, increasing the chance of bringing novel variation into the region and supporting the theory that anthropogenic activities play a critical role in facilitating the evolution of plant pathogens in agricultural systems. Human-mediated gene flow by commercial trade and research exchange of plant materials has also been documented in many other pathogens (Meng et al., 2018). Taken together, these observations indicate that appropriate quarantine is an essential strategy to reduce the evolutionary potential of plant pathogens and support agricultural and ecological sustainability (Zhan et al., 2015; Burdon and Zhan, 2020).

The wild-type Avr4 protein comprises an SP and RxLR-dEER domain in N-terminal and 3 W and 1 Y domains in C-terminal (Figure 2A). The C-terminal, particularly the W2 domain, is essential to elicit host immune response (Van Poppel et al., 2009). Frameshifts caused by the single base-pair deletions in the current (Figure 2) and previous (Van Poppel et al., 2008) studies generate two premature stop codons, truncating almost the entire C-terminal of the effector protein starting from the 92/97 amino acids before the W1 domain. Due to this fact, it is proposed that Avr4 is a pseudogene (Vleeshouwers et al., 2011) and, therefore, is expected to be exempt from natural selection. However, we have several lines of evidence to argue against the hypothesis: First, we found marginally but significantly higher *F_ST_* in the Avr4 sequences than *F_ST_* in SSR neutral markers loci in the majority of pairwise comparisons (Table 2), suggesting the Avr4 gene is under selection, but the selection is rather weak (Zhan et al., 2005). Second, isolation-by-distance analysis reveals no association between pairwise population differentiation in the neutral SSR marker loci and physical distance but a significant (but again marginal) association between pairwise population differentiation in the Avr4 sequences and physical distance (Figure 5), consistent with the scenarios that constant gene flow caused by natural or human-mediated dispersals prevents the pathogen from reaching drift-migration equilibrium in the neutral genomes but deterministic event associated with natural selection, although being weak, for local adaptation works synergistically with a random event to facilitate the realization of the equilibrium in the effector gene (Meng et al., 2018). Third, artificially truncating the C-terminal of a wild-type Avr4 by molecular manipulation also resulted in a comparable reaction, i.e., virulent phenotype, with potato plants carrying the R4 gene (Van Poppel et al., 2008, 2009). Therefore, we believe that the corresponding R4 protein can recognize the Avr4 protein encoded by the wild-type sequence, which has an intact length, whereas the virulent form with the C-terminal truncation cannot be recognized by R4, as documented previously (van Poppel et al., 2008). Thus C-terminal truncation, which happened in all Avr4 sequences sampled in six distinct geographical locations, is a mutation mechanism the pathogen equips to circumvent the immune response of potato plants.

Our results suggest that the Avr4 gene could rapidly evolve to virulent type by protein truncation. Due to this fact, we cannot unequivocally distinguish whether the natural selection inferred by the comparative analyses discussed earlier of population genetic differentiation and isolate by distance reflects a current event driven by the deployment of R4 or the evolutionary past. However, by closely looking at the sequence characteristic of the genes, particularly the untranslated parts, we argue that natural selection is likely a current event driven by the agricultural deployment of the resistance gene for late blight management. Although early termination was found, the nucleotide sequences of the current study were highly similar to the reference sequence downloaded from NCBI (Figure 2D). Furthermore, wild type with fully translated protein was detected recently outside China by other laboratories (e.g., Van Poppel et al., 2008). Intact nucleotide domains corresponding to Y motif of the wild type exist in most of the sequences (Figure 2). In addition to deletion, another potential premature stop codon generated by point mutation was found in many of the sequences. This was found in 104 (91%) of the untranslated parts of the nucleotide sequences (Figure 2C). The sequences without the point mutation (Figure 2B) were genetically closer to the reference than those with the mutation (Figure 2C). These results suggest that the ΔT^194^ deletion may be a recent event occurring at a later stage than the point mutation in the nucleotide 376 l, although both mutations lead to early termination and further suggest the selection is a recent event. The results also indicate that early termination to truncate protein is a common phenomenon in the Avr4 gene and can be induced by multiple mechanisms of point mutation and/or deletion.

The finding of heterogeneous distribution in the Avr4 sequences is expected to be generated by host selection associated with the spatial deployment of potato varieties with different resistance. Indeed, the potato varieties used over the past decades in China vary tremendously in resistant backgrounds. Some of these varieties such as Epoka and Mira and their offspring such as Yunshu 505 and Kexing No. 2 carry R4, whereas many other varieties carry other resistant genes such as R3, R8, R9, etc. (Van Poppel et al., 2009; Rietman et al., 2012; Li et al., 2018). In addition, climatic factors such as air temperature may also contribute to the heterogeneity. Temperature is one of the most important climatic factors critically impacting many evolutionary features of insects, plants, and microbes, including mutation rate (Banta et al., 1998; Li et al., 2003; Lovato et al., 2009; Chu et al., 2018). It has been reported that the mutation rate was increased under higher temperatures in bacteria and insects (Berger et al., 2017; Chu et al., 2018), and the population genetic structure and evolution of many fungal pathogens were influenced by local air temperature (Stefansson et al., 2013). In *P. infestans*, we previously reported that local air temperature affects the gene expression (unpublished data), virulence frequency, pathogenicity, fungicide sensitivity, intrinsic growth rate, and niche breadth of the pathogen (Qin et al., 2016; Wu et al., 2016; Yang et al., 2016, 2020). Here, we hypothesize that local air temperature could also be one of the factors influencing the evolution of effector genes in *P. infestans*, as indicated by a marginal but significant association of annual mean temperature in the collection sites with nucleotide diversity and/or population differentiation of Avr2 (Yang et al., 2020), Avr3a (Yang et al., 2018), and Avr4 (Table 1; Figure 3). Although it is usually difficult to distinguish “cause” from “effect” by the correlation analysis, in this particular case, we believe that temperature is the cause affecting sequence characters of AVR4, not the other way. The weak associations between local temperature and sequence characters of *P. infestans* effectors in the current and previous studies may attribute to fewer populations (collection sites) involved. Future studies should cover populations originating from many locations (>20) varying in thermal zones or use an experimental evolution approach (e.g., Wu et al., 2020) to confirm the finding.

## Conclusion

Our results provide important insights into the evolutionary causes and processes of effector genes and host-pathogen arms race. Together with the previous publications (Yang et al., 2018, 2020), our results show that *P. infestans* has evolved diverse mechanisms to escape R gene recognition, ranging from differential transcription and structure disordering in Avr2 (Gilroy et al., 2011; Yang et al., 2020), single amino acid change in Avr3a (Yang et al., 2018), and sequence truncation in Avr4, and similar phenomenon may exist in other pathogens as well. Furthermore, abiotic factors such as air temperature may also contribute to the population dynamics and evolution of effector genes. In practice, host resistance is an environment-friendly approach to manage plant diseases, but the rapid evolution of effector genes empowers plant pathogen propensity to quickly escape host defense systems, greatly threatening agricultural and ecological sustainability. To achieve sustainable plant disease management, adaptive disease management programs based on the principles of evolutionary ecology (Zhan et al., 2014, 2015), such as through spatiotemporal deployment of resistance genes (Zhan et al., 2002; Yang et al., 2019) and other available pathogen mitigation arsenals (Karlsson Green et al., 2020), are necessary. This is particularly important in the current era of climate change, such as global warming, which not only exerts eminent and short-term influences on the epidemics of plant diseases but also produces last, long-term impacts on the evolution of plant pathogens (Zhan and McDonald, 2011; Yang et al., 2016; Wu et al., 2020). Regarding Avr4, the mutation in this effector has completely rendered the corresponding R4 in potato, and cultivars with this particular resistance gene alone are unable to control the late blight disease effectively.

## Data Availability Statement

The sequences presented in this study can be found in NCBI with accession numbers of MW774780-MW774893.

## Author Contributions

JZ and L-NY conceived and designed the experiments. AW, Y-PW, and ON collected the pathogen isolates. L-NY, AW, Y-PW, ON, S-TL, W-YL, and YL generated the data. AW, Y-PW, ON, L-NY, and JZ analyzed and interpreted the data. L-NY, Z-HW, and G-DL supervised the project. AW, L-NY, and JZ wrote the paper. All authors contributed to the article and approved the submitted version.

### Conflict of Interest

The authors declare that the research was conducted in the absence of any commercial or financial relationships that could be construed as a potential conflict of interest.

## Footnotes

^1^http://www.ualberta.ca/~fyeh/popgene_download.html

^2^http://popart.otago.ac.nz

^3^http://www.worldclimate.com/

## References

[ref1] Asai S. Shirasu K. (2015). Plant cells under siege: plant immune system versus pathogen effectors. Curr. Opin. Plant Biol. 28, 1–8. 10.1016/j.pbi.2015.08.008, PMID: 26343014

[ref2] Banta L. M. Bohne J. Lovejoy S. D. Dostal K. (1998). Stability of the *Agrobacterium tumefaciens* VirB10 protein is modulated by growth temperature and periplasmic osmoadaption. J. Bacteriol. 180, 6597–6606. 10.1128/JB.180.24.6597-6606.1998, PMID: 9852004 PMC107763

[ref3] Berger D. Stångberg J. Grieshop K. Martinossi-Allibert I. Arnqvist G. (2017). Temperature effects on life-history trade-offs, germline maintenance and mutation rate under simulated climate warming. Proc. Biol. Sci. 284:20171721. 10.1098/rspb.2017.1721, PMID: 29118134 PMC5698646

[ref4] Birch P. R. Armstrong M. Bos J. Boevink P. Gilroy E. M. Taylor R. M.. (2009). Towards understanding the virulence functions of RXLR effectors of the oomycete plant pathogen *Phytophthora infestans*. J. Exp. Bot. 60, 1133–1140. 10.1093/jxb/ern353, PMID: 19204033

[ref5] Bos J. I. Armstrong M. Whisson S. C. Torto T. A. Ochwo M. Birch P. R.. (2003). Intraspecific comparative genomics to identify avirulence genes from *Phytophthora*. New Phytol. 159, 63–72. 10.1046/j.1469-8137.2003.00801.x 33873680

[ref6] Burdon J. J. Zhan J. (2020). Climate change and disease in plant communities. PLoS Biol. 18:e3000949. 10.1371/journal.pbio.3000949, PMID: 33232314 PMC7685433

[ref7] Cárdenas M. Grajales A. Sierra R. Rojas A. González-Almario A. Vargas A.. (2011). Genetic diversity of *Phytophthora infestans* in the northern Andean region. BMC Genet. 12:23. 10.1186/1471-2156-12-23, PMID: 21303555 PMC3046917

[ref8] Cheng C. Gao X. Feng B. Sheen J. Shan L. He P. (2013). Plant immune response to pathogens differs with changing temperatures. Nat. Commun. 4, 1–9. 10.1038/ncomms3530, PMID: 24067909 PMC3901997

[ref9] Chu X. L. Zhang B. W. Zhang Q. G. Zhu B. R. Lin K. Zhang D. Y. (2018). Temperature responses of mutation rate and mutational spectrum in an *Escherichia coli* strain and the correlation with metabolic rate. BMC Evol. Biol. 18:126. 10.1186/s12862-018-1252-8, PMID: 30157765 PMC6116381

[ref10] Excoffier L. Lischer H. E. (2010). Arlequin suite ver 3.5: a new series of programs to perform population genetics analyses under Linux and windows. Mol. Ecol. Resour. 10, 564–567. 10.1111/j.1755-0998.2010.02847.x, PMID: 21565059

[ref11] Faostat F. (2017). QC. Available at: http://www.fao.org/faostat/en/#data (Accessed January 2018).

[ref12] Flier W. G. Grünwald N. J. Kroon L. P. Sturbaum A. K. van den Bosch T. B. Garay-Serrano E.. (2003). The population structure of *Phytophthora infestans* from the Toluca Valley of Central Mexico suggests genetic differentiation between populations from cultivated potato and wild *Solanum* spp. Phytopathology 93, 382–390. 10.1094/PHYTO.2003.93.4.382, PMID: 18944351

[ref13] Flor H. (1942). Inheritance of pathogenicity in Melampsora lini. Phytopathology 32, 653–669. 10.1094/Phyto-32-653 40934406

[ref14] Fry W. (2008). *Phytophthora infestans*: the plant (and R gene) destroyer. Mol. Plant Pathol. 9, 385–402. 10.1111/j.1364-3703.2007.00465.x, PMID: 18705878 PMC6640234

[ref15] Gao F. Zou W. Xie L. Zhan J. (2017). Adaptive evolution and demographic history contribute to the divergent population genetic structure of potato virus Y between China and Japan. Evol. Appl. 10, 379–390. 10.1111/eva.12459, PMID: 28352297 PMC5367074

[ref16] Gilroy E. M. Breen S. Whisson S. C. Squires J. Hein I. Kaczmarek M.. (2011). Presence/absence, differential expression and sequence polymorphisms between PiAVR2 and PiAVR2-like in *Phytophthora infestans* determine virulence on R2 plants. New Phytol. 191, 763–776. 10.1111/j.1469-8137.2011.03736.x, PMID: 21539575

[ref17] Goritschnig S. Krasileva K. V. Dahlbeck D. Staskawicz B. J. (2012). Computational prediction and molecular characterization of an oomycete effector and the cognate *Arabidopsis* resistance gene. PLoS Genet. 8:e1002502. 10.1371/journal.pgen.1002502, PMID: 22359513 PMC3280963

[ref18] Haas B. J. Kamoun S. Zody M. C. Jiang R. H. Handsaker R. E. Cano L. M.. (2009). Genome sequence and analysis of the Irish potato famine pathogen *Phytophthora infestans*. Nature 461, 393–398. 10.1038/nature08358, PMID: 19741609

[ref19] Harry M. Dupont L. Quartier M. Diotaiuti L. Walter A. Romana C. (2009). New perspectives for population genetics of Chagas'disease vectors in the Northeastern Brazil: isolation of polymorphic microsatellite markers in *Triatoma brasiliensis*. Infect. Genet. Evol. 9, 633–637. 10.1016/j.meegid.2009.03.008, PMID: 19460330

[ref20] Hein I. Gilroy E. M. Armstrong M. R. Birch P. R. (2009). The zig-zag-zig in oomycete–plant interactions. Mol. Plant Pathol. 10, 547–562. 10.1111/j.1364-3703.2009.00547.x, PMID: 19523107 PMC6640229

[ref21] Jiang R. H. Tripathy S. Govers F. Tyler B. M. (2008). RXLR effector reservoir in two *Phytophthora* species is dominated by a single rapidly evolving superfamily with more than 700 members. Proc. Natl. Acad. Sci. 105, 4874–4879. 10.1073/pnas.0709303105 18344324 PMC2290801

[ref22] Jones J. D. Dangl J. L. (2006). The plant immune system. Nature 444, 323–329. 10.1038/nature05286, PMID: 17108957

[ref23] Kamoun S. (2006). A catalogue of the effector secretome of plant pathogenic oomycetes. Annu. Rev. Phytopathol. 44, 41–60. 10.1146/annurev.phyto.44.070505.143436, PMID: 16448329

[ref24] Karasov T. L. Horton M. W. Bergelson J. (2014). Genomic variability as a driver of plant–pathogen coevolution? Curr. Opin. Plant Biol. 18, 24–30. 10.1016/j.pbi.2013.12.003, PMID: 24491596 PMC4696489

[ref25] Karlsson Green K. Stenberg J. A. Lankinen Å. (2020). Making sense of integrated pest management (IPM) in the light of evolution. Evol. Appl. 13, 1791–1805. 10.1111/eva.13067, PMID: 32908586 PMC7463341

[ref26] Knapova G. Gisi U. (2002). Phenotypic and genotypic structure of *Phytophthora infestans* populations on potato and tomato in France and Switzerland. Plant Pathol. 51, 641–653. 10.1046/j.1365-3059.2002.00750.x

[ref27] Lamour K. Kamoun S. (2009). Oomycete Genetics and Genomics: Diversity, Interactions and Research Tools. Hoboken, New Jersey: John Wiley & Sons.

[ref28] Lawrence I. Lin K. (1989). A concordance correlation coefficient to evaluate reproducibility. Biometrics 45, 255–268. 10.2307/2532051 2720055

[ref29] Lees A. Wattier R. Shaw D. Sullivan L. Williams N. Cooke D. (2006). Novel microsatellite markers for the analysis of *Phytophthora infestans* populations. Plant Pathol. 55, 311–319. 10.1111/j.1365-3059.2006.01359.x

[ref30] Li Y. Cole K. Altman S. (2003). The effect of a single, temperature-sensitive mutation on global gene expression in *Escherichia coli*. RNA 9, 518–532. 10.1261/rna.2198203, PMID: 12702811 PMC1370418

[ref31] Li X. Xu J. Duan S. Bian C. Hu J. Shen H.. (2018). Pedigree-based deciphering of genome-wide conserved patterns in an elite potato parental line. Front. Plant Sci. 9:690. 10.3389/fpls.2018.00690, PMID: 29875792 PMC5974212

[ref32] Librado P. Rozas J. (2009). DnaSP v5: a software for comprehensive analysis of DNA polymorphism data. Bioinformatics 25, 1451–1452. 10.1093/bioinformatics/btp187, PMID: 19346325

[ref33] Lovato T. A. L. Adams M. M., Baker, P. W., and RCripps R. M. (2009). A molecular mechanism of temperature sensitivity for mutations affecting the drosophila muscle regulator myocyte enhancer factor-2. Genetics 183, 107–117. 10.1534/genetics.109.105056, PMID: 19564485 PMC2746136

[ref34] Lurwanu Y. Wang Y. P. Wu E. J. He D. C. Waheed A. Nkurikiyimfura O.. (2021). Increasing temperature elevates the variation and spatial differentiation of pesticide tolerance in a plant pathogen. Evol. Appl. 14, 1274–1285. 10.1111/eva.13197 34025767 PMC8127700

[ref35] Martin F. N. (2000). Phylogenetic relationships among some *Pythium* species inferred from sequence analysis of the mitochondrially encoded cytochrome oxidase II gene. Mycologia 92, 711–727. 10.1080/00275514.2000.12061211 21156613

[ref36] Martin D. P. Murrell B. Golden M. Khoosal A. Muhire B. (2015). RDP4: detection and analysis of recombination patterns in virus genomes. Virus Evol. 1:vev003. 10.1093/ve/vev003, PMID: 27774277 PMC5014473

[ref37] Mboup M. Bahri B. Leconte M. De Vallavieille-Pope C. Kaltz O. Enjalbert J. (2012). Genetic structure and local adaptation of European wheat yellow rust populations: the role of temperature-specific adaptation. Evol. Appl. 5, 341–352. 10.1111/j.1752-4571.2011.00228.x, PMID: 25568055 PMC3353355

[ref38] McDonald B. A. Linde C. (2002). The population genetics of plant pathogens and breeding strategies for durable resistance. Euphytica 124, 163–180. 10.1023/A:1015678432355

[ref39] Meng J.-W. He D.-C. Zhu W. Yang L.-N. Wu E.-J. Xie J.-H.. (2018). Human-mediated gene flow contributes to metapopulation genetic structure of the pathogenic fungus *Alternaria alternata* from potato. Front. Plant Sci. 9:198. 10.3389/fpls.2018.00198, PMID: 29497439 PMC5818430

[ref40] Menna A. Nguyen D. Guttman D. S. Desveaux D. (2015). Elevated temperature differentially influences effector-triggered immunity outputs in *Arabidopsis*. Front. Plant Sci. 6:995. 10.3389/fpls.2015.00995, PMID: 26617631 PMC4637416

[ref41] Möller M. Stukenbrock E. H. (2017). Evolution and genome architecture in fungal plant pathogens. Nat. Rev. Microbiol. 15:756. 10.1038/nrmicro.2017.76, PMID: 28781365

[ref42] Qin C.-F. He M.-H. Chen F.-P. Zhu W. Yang L.-N. Wu E.-J.. (2016). Comparative analyses of fungicide sensitivity and SSR marker variations indicate a low risk of developing azoxystrobin resistance in *Phytophthora infestans*. Sci. Rep. 6, 1–10. 10.1038/srep20483 26853908 PMC4745062

[ref43] Rietman H. Bijsterbosch G. Cano L. M. Lee H.-R. Vossen J. H. Jacobsen E.. (2012). Qualitative and quantitative late blight resistance in the potato cultivar Sarpo Mira is determined by the perception of five distinct RXLR effectors. Mol. Plant-Microbe Interact. 25, 910–919. 10.1094/MPMI-01-12-0010-R, PMID: 22414442

[ref44] Schornack S. Huitema E. Cano L. M. Bozkurt T. O. Oliva R. Van Damme M.. (2009). Ten things to know about oomycete effectors. Mol. Plant Pathol. 10, 795–803. 10.1111/j.1364-3703.2009.00593.x, PMID: 19849785 PMC6640533

[ref45] Shan W. Cao M. Leung D. Tyler B. M. (2004). The Avr1b locus of *Phytophthora sojae* encodes an elicitor and a regulator required for avirulence on soybean plants carrying resistance gene Rps 1b. Mol. Plant-Microbe Interact. 17, 394–403. 10.1094/MPMI.2004.17.4.394, PMID: 15077672

[ref46] Stefańczyk E. Brylińska M. Brurberg M. B. Naerstad R. Elameen A. Sobkowiak S.. (2018). Diversity of Avr-vnt1 and AvrSmira1 effector genes in polish and Norwegian populations of *Phytophthora infestans*. Plant Pathol. 67, 1792–1802. 10.1111/ppa.12875

[ref47] Stefansson T. S. McDonald B. A. Willi Y. (2013). Local adaptation and evolutionary potential along a temperature gradient in the fungal pathogen *Rhynchosporium commune*. Evol. Appl. 6, 524–534. 10.1111/eva.12039, PMID: 23745143 PMC3673479

[ref48] Stukenbrock E. H. McDonald B. A. (2009). Population genetics of fungal and oomycete effectors involved in gene-for-gene interactions. Mol. Plant-Microbe Interact. 22, 371–380. 10.1094/MPMI-22-4-0371, PMID: 19271952

[ref49] Tamura K. Peterson D. Peterson N. Stecher G. Nei M. Kumar S. (2011). MEGA5: molecular evolutionary genetics analysis using maximum likelihood, evolutionary distance, and maximum parsimony methods. Mol. Biol. Evol. 28, 2731–2739. 10.1093/molbev/msr121, PMID: 21546353 PMC3203626

[ref50] Tanaka S. Schweizer G. Rössel N. Fukada F. Thines M. Kahmann R. (2019). Neofunctionalization of the secreted Tin2 effector in the fungal pathogen *Ustilago maydis*. Nat. Microbiol. 4, 251–257. 10.1038/s41564-018-0304-6, PMID: 30510169

[ref51] Thrall P. H. Barrett L. G. Dodds P. N. Burdon J. J. (2016). Epidemiological and evolutionary outcomes in gene-for-gene and matching allele models. Front. Plant Sci. 6:1084. 10.3389/fpls.2015.01084, PMID: 26779200 PMC4703789

[ref52] Tian M. Win J. Song J. van der Hoorn R. van der Knaap E. Kamoun S. (2007). A *Phytophthora infestans* cystatin-like protein targets a novel tomato papain-like apoplastic protease. Plant Physiol. 143, 364–377. 10.1104/pp.106.090050, PMID: 17085509 PMC1761951

[ref53] Tyler B. M. Tripathy S. Zhang X. Dehal P. Jiang R. H. Aerts A.. (2006). *Phytophthora* genome sequences uncover evolutionary origins and mechanisms of pathogenesis. Science 313, 1261–1266. 10.1126/science.1128796, PMID: 16946064

[ref54] Van Der Biezen E. A. Jones J. D. (1998). Plant disease-resistance proteins and the gene-for-gene concept. Trends Biochem. Sci. 23, 454–456. 10.1016/S0968-0004(98)01311-5, PMID: 9868361

[ref55] Van Poppel P. M. Guo J. van de Vondervoort P. J. Jung M. W. Birch P. R. Whisson S. C.. (2008). The *Phytophthora infestans* avirulence gene Avr4 encodes an RXLR-dEER effector. Mol. Plant-Microbe Interact. 21, 1460–1470. 10.1094/MPMI-21-11-1460, PMID: 18842095

[ref56] Van Poppel P. M. Jiang R. H. Śliwka J. Govers F. (2009). Recognition of *Phytophthora infestans* Avr4 by potato R4 is triggered by C-terminal domains comprising W motifs. Mol. Plant Pathol. 10, 611–620. 10.1111/j.1364-3703.2009.00556.x, PMID: 19694952 PMC6640270

[ref57] Velásquez A. C. Castroverde C. D. M. He S. Y. (2018). Plant–pathogen warfare under changing climate conditions. Curr. Biol. 28, R619–R634. 10.1016/j.cub.2018.03.054, PMID: 29787730 PMC5967643

[ref58] Vleeshouwers V. G. Raffaele S. Vossen J. H. Champouret N. Oliva R. Segretin M. E.. (2011). Understanding and exploiting late blight resistance in the age of effectors. Annu. Rev. Phytopathol. 49, 507–531. 10.1146/annurev-phyto-072910-095326, PMID: 21663437

[ref59] Wang X. Boevink P. McLellan H. Armstrong M. Bukharova T. Qin Z.. (2015). A host KH RNA-binding protein is a susceptibility factor targeted by an RXLR effector to promote late blight disease. Mol. Plant 8, 1385–1395. 10.1016/j.molp.2015.04.012, PMID: 25936676 PMC4560694

[ref60] Wang Q. Li T. Zhong C. Luo S. Xu K. Gu B.. (2019a). Small RNAs generated by bidirectional transcription mediate silencing of RXLR effector genes in the oomycete *Phytophthora sojae*. Phytopathol. Res. 1:18. 10.1186/s42483-019-0026-6

[ref61] Wang S. McLellan H. Bukharova T. He Q. Murphy F. Shi J.. (2019b). *Phytophthora infestans* RXLR effectors act in concert at diverse subcellular locations to enhance host colonization. J. Exp. Bot. 70, 343–356. 10.1093/jxb/ery360 30329083 PMC6305197

[ref62] Wang Y.-P. Xie J.-H. Wu E.-J. Yahuza L. Duan G.-H. Shen L.-L.. (2020). Lack of gene flow between *Phytophthora infestans* populations of two neighboring countries with the largest potato production. Evol. Appl. 13, 318–329. 10.1111/eva.12870 31993079 PMC6976962

[ref63] Win J. Morgan W. Bos J. Krasileva K. V. Cano L. M. Chaparro-Garcia A.. (2007). Adaptive evolution has targeted the C-terminal domain of the RXLR effectors of plant pathogenic oomycetes. Plant Cell 19, 2349–2369. 10.1105/tpc.107.051037, PMID: 17675403 PMC2002621

[ref64] Wu Y. Jiang J. Gui C. (2012). Low genetic diversity of *Phytophthora infestans* population in potato in North China. Afr. J. Biotechnol. 11, 15636–15642. 10.5897/AJB12.484

[ref65] Wu E.-J. Wang Y.-P. Shen L.-L. Yahuza L. Tian J.-C. Yang L.-N.. (2019). Strategies of *Phytophthora infestans* adaptation to local UV radiation conditions. Evol. Appl. 12, 415–424. 10.1111/eva.12722 30828364 PMC6383706

[ref66] Wu E.-J. Wang Y.-P. Yahuza L. He M.-H. Sun D.-L. Huang Y.-M.. (2020). Rapid adaptation of the Irish potato famine pathogen *Phytophthora infestans* to changing temperature. Evol. Appl. 13, 768–780. 10.1111/eva.12899, PMID: 32211066 PMC7086108

[ref67] Wu E.-J. Yang L.-N. Zhu W. Chen X.-M. Shang L.-P. Zhan J. (2016). Diverse mechanisms shape the evolution of virulence factors in the potato late blight pathogen *Phytophthora infestans* sampled from China. Sci. Rep. 6:26182. 10.1038/srep26182 27193142 PMC4872137

[ref68] Yang L.-N. He M.-H. Ouyang H.-B. Zhu W. Pan Z.-C. Sui Q.-J.. (2019). Cross-resistance of the pathogenic fungus *Alternaria alternata* to fungicides with different modes of action. BMC Microbiol. 19:205. 10.1186/s12866-019-1574-8, PMID: 31477005 PMC6720428

[ref69] Yang L.-N. Liu H. Duan G.-H. Huang Y.-M. Liu S.-T. Fang Z.-G.. (2020). *Phytophtora infestans* AVR2 effector escapes R2 recognition through effector disordering. Mol. Plant-Microbe Interact. 33, 921–931. 10.1094/MPMI-07-19-0179-R, PMID: 32212906

[ref70] Yang L.-N. Ouyang H.-B. Fang Z.-G. Zhu W. Wu E.-J. Luo G.-H.. (2018). Evidence for intragenic recombination and selective sweep in an effector gene of *Phytophthora infestans*. Evol. Appl. 11, 1342–1353. 10.1111/eva.12629, PMID: 30151044 PMC6099815

[ref71] Yang L. Zhu W. Wu E. J. Yang C. Thrall P. H. Burdon J. J.. (2016). Trade-offs and evolution of thermal adaptation in the Irish potato famine pathogen *Phytophthora infestans*. Mol. Ecol. 25, 4047–4058. 10.1111/mec.13727, PMID: 27288627

[ref72] Yin J. Gu B. Huang G. Tian Y. Quan J. Lindqvist-Kreuze H.. (2017). Conserved RXLR effector genes of *Phytophthora infestans* expressed at the early stage of potato infection are suppressive to host defense. Front. Plant Sci. 8:2155. 10.3389/fpls.2017.02155, PMID: 29312401 PMC5742156

[ref73] Zhan J. Linde C. C. Jürgens T. Merz U. Steinebrunner F. McDonald B. A. (2005). Variation for neutral markers is correlated with variation for quantitative traits in the plant pathogenic fungus *Mycosphaerella graminicola*. Mol. Ecol. 14, 2683–2693. 10.1111/j.1365-294X.2005.02638.x, PMID: 16029470

[ref74] Zhan J. McDonald B. A. (2011). Thermal adaptation in the fungal pathogen *Mycosphaerella graminicola*. Mol. Ecol. 20, 1689–1701. 10.1111/j.1365-294X.2011.05023.x, PMID: 21395890

[ref75] Zhan J. Mundt C. C. Hoffer M. McDonald B. A. (2002). Local adaptation and effect of host genotype on the rate of pathogen evolution: an experimental test in a plant pathosystem. J. Evol. Biol. 15, 634–647. 10.1046/j.1420-9101.2002.00428.x

[ref76] Zhan J. Pettway R. E. McDonald B. A. (2003). The global genetic structure of the wheat pathogen *Mycosphaerella graminicola* is characterized by high nuclear diversity, low mitochondrial diversity, regular recombination, and gene flow. Fungal Genet. Biol. 38, 286–297. 10.1016/S1087-1845(02)00538-8, PMID: 12684018

[ref77] Zhan J. Thrall P. H. Burdon J. J. (2014). Achieving sustainable plant disease management through evolutionary principles. Trends Plant Sci. 19, 570–575. 10.1016/j.tplants.2014.04.010, PMID: 24853471

[ref78] Zhan J. Thrall P. H. Papaïx J. Xie L. Burdon J. J. (2015). Playing on a pathogen's weakness: using evolution to guide sustainable plant disease control strategies. Annu. Rev. Phytopathol. 53, 19–43. 10.1146/annurev-phyto-080614-120040, PMID: 25938275

[ref79] Zhou J.-L. Xu J. Jiao A.-G. Yang L. Chen J. Callac P.. (2019). Patterns of PCR amplification artifacts of the fungal barcode marker in a hybrid mushroom. Front. Microbiol. 10:2686. 10.3389/fmicb.2019.02686, PMID: 31803173 PMC6877668

[ref80] Zhu W. Shen L.-L. Fang Z.-G. Yang L.-N. Zhang J.-F. Sun D.-L.. (2016). Increased frequency of self-fertile isolates in *Phytophthora infestans* may attribute to their higher fitness relative to the A1 isolates. Sci. Rep. 6, 1–10. 10.1038/srep29428 27384813 PMC4935937

[ref81] Zhu W. Yang L.-N. Wu E.-J. Qin C.-F. Shang L.-P. Wang Z.-H.. (2015). Limited sexual reproduction and quick turnover in the population genetic structure of *Phytophthora infestans* in Fujian, China. Sci. Rep. 5:10094. 10.1038/srep10094 25970264 PMC4429539

